# Inverted Yoga and Near Syncope: An Unusual Diagnosis of Right Ventricular Myxoma

**DOI:** 10.1155/2018/9231256

**Published:** 2018-10-01

**Authors:** Wadie David, Narmeen Rehman, Robby Singh, Shukri David

**Affiliations:** Providence Hospital, Southfield, MI, USA

## Abstract

Primary cardiac tumors are extremely rare and are difficult to diagnose. Although usually benign in nature, myxomas require surgical resection due to their increased risk of embolic and cardiac complications, with the timing of resection dependent on the presentation and size of the tumor. However, if diagnosed early, patients with primary benign cardiac tumors have excellent prognosis following surgery. Therefore, a high index of suspicion and a wide differential diagnosis are very important in detecting rare conditions that can affect otherwise healthy individuals. We present an uncommon case of a right ventricular myxoma that was discovered when the patient was performing inverted yoga and experienced a near syncopal episode. Patient subsequently underwent an echocardiographic evaluation and was found to have a right ventricular myxoma that was excised. Although recurrence is rare, it is important for physicians to remain vigilant and continue careful and consistent follow-up for patients with a history of a cardiac myxoma.

## 1. Introduction

Primary cardiac tumors are very rare with an incidence rate of between 0.0017 and. 19% reported on autopsies of unselected patients and most are benign [1]. The vast majority, roughly 70–80%, are found in the left atrium and 10–20% in the right atrium [2]. Right ventricular myxomas are rarer still, with an incidence of only 2–4% [3]. Patients are typically asymptomatic and the malignancies are discovered either incidentally or when embolization or myocardial infiltration has occurred, which may lead to a rapid demise. We herein describe an extremely rare case of a right ventricular myxoma with an unusual clinical presentation.

## 2. Case Presentation

**Figure 1:**
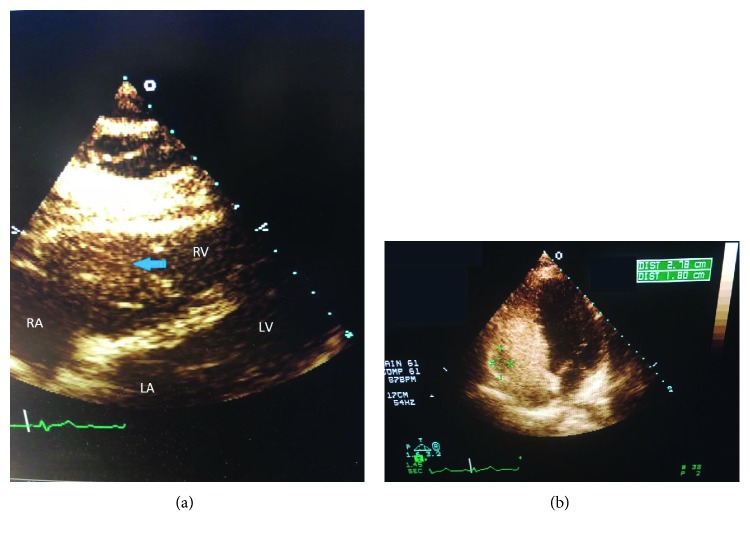
(a) Transthoracic echocardiogram with right ventricular myxoma (arrow). (b) Transthoracic echocardiogram with agitated saline revealing a right ventricular myxoma measuring 2.78 cm × 1.8 cm.

A 51-year-old previously healthy and physically active woman presented as an outpatient with complaints of lightheadedness and dizziness. In addition to her activities of daily living, the patient plays competitive tennis without any limitations or symptoms. Recently, while doing aerial yoga, she felt lightheaded and experienced a presyncopal episode, specifically while hanging upside down and performing hand stands. On physical exam, vital signs were normal and the exam was unremarkable except for the cardiac exam. The precordium was quiet with no displacement of the point of maximal impulse. There was a grade III/VI systolic murmur at the left sternal border and the electrocardiogram revealed a left bundle branch block. Subsequently, a transthoracic echocardiogram was performed and revealed a mass in the right ventricle attached to the posterior wall and prolapsing into the right ventricular outflow tract (RVOT) in systole (Figure 1).

**Figure 2:**
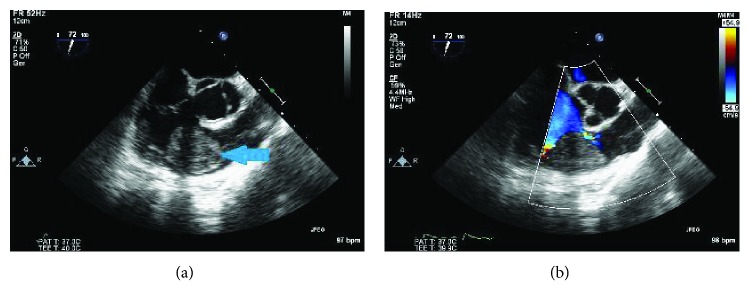
(a) RV myxoma on TEE (arrow). (b) Color flow around RV myoxoma on TEE.

**Figure 3:**
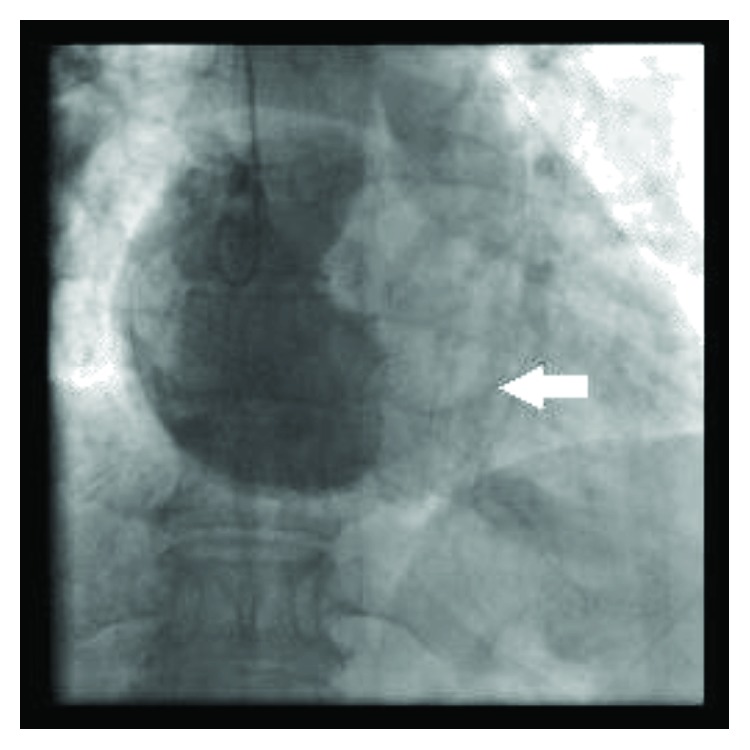
RV mass appreciated on cardiac catheterization.

**Figure 4:**
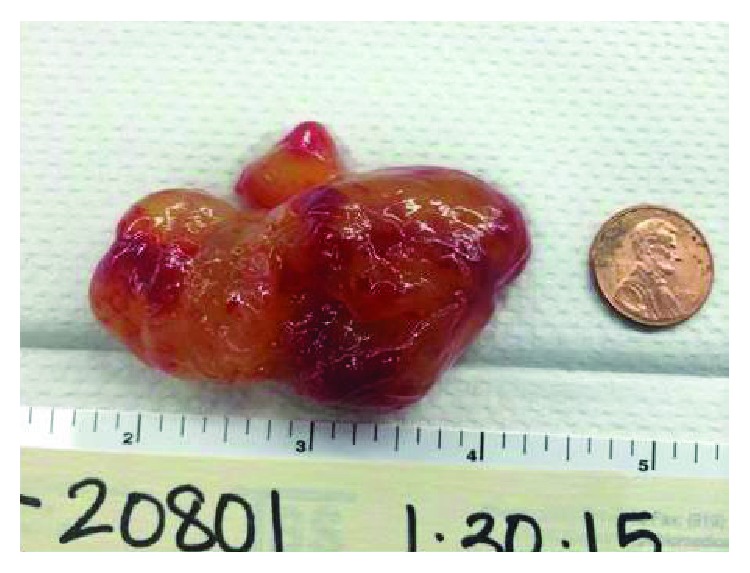
RV myxoma excised.

**Figure 5:**
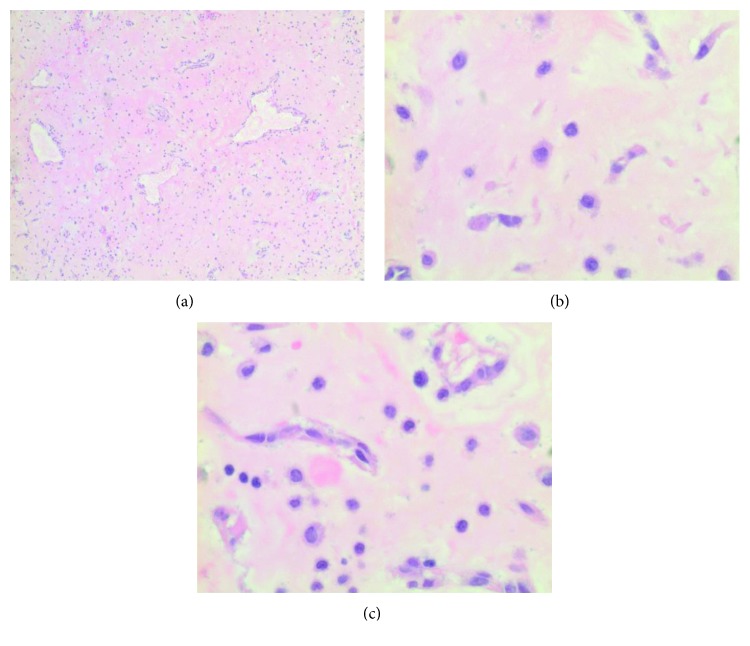
The tumor is comprised of spindled or stellate cells with eosinophilic cytoplasm and uniform, round nuclei with inconspicuous nucleoli in a background of myxoid stroma. Chronic inflammatory cells including hemosiderin-laden macrophages are also present. The cellularity is variable; however, no foci of increased cellularity marked nuclear pleomorphism or increased mitotic activity seen.

**Figure 6:**
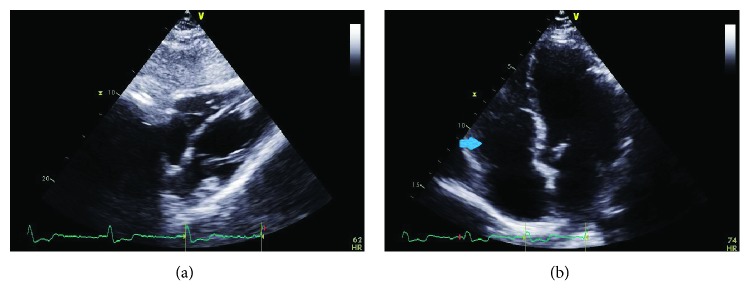
(a) Subcostal view on transthoracic echocardiogram two years postprocedure without evidence of recurrence of RV myxoma. (b) Apical view from the same study (arrow: right ventricle).

Next, a transesophageal echocardiogram was performed to better characterize the mass, which measured 2.6 cm × 4.1 cm and was mobile and prolapsed into the RVOT during systole resulting in a mild dynamic obstruction (Figure 2(a)). Color Doppler revealed turbulent flow in the RVOT and pressure gradient measurements revealed a gradient of 26 mmHg across the RVOT while the patient was heavily sedated and in a supine position (Figure 2(b)). We suspect that with aerial yoga, and the resulting upside-down suspension, the gradient would be higher. Therefore, the obstruction would be enhanced, resulting in decreased cardiac output and cerebral hypoperfusion, which could explain the presenting symptoms of lightheadedness and dizziness. The patient was referred to cardiothoracic surgery for further evaluation and underwent a cardiac catheterization prior to thoracotomy which also showed evidence of a RV mass (Figure 3). A thoracotomy with excision of the mass was performed (Figure 4), and pathological examination revealed the mass as a myxoma (Figure 5). The patient had an uneventful recovery and was discharged home. She has been continued to follow up regularly two years postresection of the myxoma and continues to do well. She continues to experience no limitations in performing her activities of daily living nor while playing competitive tennis or performing aerial yoga. Repeat echocardiogram two years postresection did not reveal any evidence of recurrence of the myxoma (Figures 6(a) and 6(b)).

## 3. Discussion

The majority of myxomas are found in the left atrium and symptoms are typically seen when tumor growth interferes with normal cardiac function [4]. Up to 12–15% of cardiac myxomas are asymptomatic and discovered incidentally or during postmortem examination. The majority present with a combination of symptoms such as obstruction of blood flow to the neighboring chamber, thromboembolic events, hemodynamic changes, conduction disease, and otherwise nonspecific symptoms. Obstruction of blood flow can lead to impaired atrial filling, heart failure, chronic passive congestion of the lungs, and dyspnea. Obstruction can also lead to hemodynamic changes caused by systemic emboli, particularly in right-sided lesions which can propagate to the pulmonary arteries and result in obstruction or left-sided lesions causing cerebral emboli. Additionally, depending on the location of the tumor, growth can disrupt nodal or septal conduction tissue leading to heart arrhythmia and sudden cardiac death [4].

Mitral valve stenosis and regurgitation may be seen in patients with mobile left atrial tumors. On physical exam, one can also listen for a “tumor plop,” an acoustic diastolic sound that is caused by the presence of a mobile myxoma inside the atrial chamber. This is typically seen in 15% of patients and occurs when the tumor comes to rest over the mitral annulus. These tumors interfere with valve coaptation and can often be recognized by the astute clinician which may lead to further workup.

One unique characteristic of myxomas is its ability to mimic systemic autoimmune diseases. Myxomas have the capacity to secrete interleukin 6 and 8 (IL-6 and IL-8), especially IL-6, which is a pleiotropic cytokine that increases B cell differentiation and leads to increased synthesis of polyclonal immunoglobulins [4]. In addition, it is a strong hepatocyte-stimulating factor, which induces the release of acute-phase proteins [4]. IL-6 is also implicated in increased tumor growth and tumor recurrence. Theses cytokines also lead to nonspecific symptoms which are seen in about 80–85% of cases such as fatigue, lethargy, weakness, erythema, weight loss, and decreased appetite [4].

In addition, depending on the size of the tumor, many patients can present with chromic hemolytic anemia and thrombocytopenia [4]. These conditions are caused by abnormal blood flow leading to damaged RBCs as they pass over the tumor surface [4]. Diagnosis of these tumors is typically made by echocardiography which is highly sensitive and can differentiate cardiac tumors from other cardiac masses such as vegetation and thrombi [4].

While many patients present with multiple symptoms, these tumors may also be asymptomatic, making them especially difficult to diagnose. If gone undiagnosed and untreated, these can result in severe cardiac complications due to obstruction of blood flow and potential disruption of nodal conduction tissue. In our case, a right ventricular myxoma, among the rarest forms of cardiac malignancy, manifested with RVOT obstruction leading to syncope, though the outcome could have been more disastrous had the obstruction been more prolonged. For this reason, it is essential that the diagnosis is made early, and although most cases require surgical resection, recovery is often rapid. While recurrence is rare, it is important to have careful and consistent follow-up with patients who have a history of a cardiac myxoma.
